# DIAMOND (DIgital Alcohol Management ON Demand): a feasibility RCT and embedded process evaluation of a digital health intervention to reduce hazardous and harmful alcohol use recruiting in hospital emergency departments and online

**DOI:** 10.1186/s40814-018-0303-7

**Published:** 2018-06-15

**Authors:** Fiona L. Hamilton, Jo Hornby, Jessica Sheringham, Stuart Linke, Charlotte Ashton, Kevin Moore, Fiona Stevenson, Elizabeth Murray

**Affiliations:** 1 eHealth Unit, Department of Primary Care & Population Health, University College London, Upper 3rd Floor, Royal Free Campus, Rowland Hill Street, London, NW3 2PF UK; 2 Department of Applied Health Research, UCL, London, UK; 3 Camden and Islington NHS Foundation Trust, London, UK; 4 Camden and Islington Public Health, London, UK; 5 Institute for Liver and Digestive Health, UCL, London, UK

## Abstract

**Background:**

The harmful use of alcohol is a causal factor in more than 200 disease and injury conditions and leads to over 3 million deaths every year worldwide. Relatively few problem alcohol users access treatment due to stigma and lack of services. Alcohol-specific digital health interventions (DHI) may help them, but trial data comparing DHI with face-to-face treatment are lacking.

**Methods:**

We conducted a feasibility RCT of an alcohol DHI, testing recruitment, online data-collection and randomisation processes, with an embedded process evaluation. Recruitment ran from October 2015 for 12 months. Participants were adults, drinking at hazardous and harmful levels, recruited from hospital emergency departments (ED) in London or recruited online. Participants were randomised to HeLP-Alcohol, a six module DHI with weekly reminder prompts (phone, email or text message), or to face-to-face treatment as usual (TAU). Participants were invited to take part in qualitative interviews after the trial.

**Results:**

The trial website was accessed 1074 times: 420 people completed online eligibility questionnaires; 350 did not meet eligibility criteria, 51 declined to participate, and 19 were recruited and randomised. Follow-up data were collected from three participants (retention 3/19), and four agreed to be interviewed for the process evaluation. The main themes of the interviews were:

- Participants were not at equipoise. They wanted to try the website and were disappointed to be randomised to face-to-face, so they were less engaged and dropped out.
- Other reasons for drop out included not accepting that they had a drink problem; problem drinking interfering with their ability to take part in a trial or forgetting appointments; having a busy life and being randomised to TAU made it difficult to attend appointments.

**Conclusions:**

This feasibility RCT aimed to test recruitment, randomisation, retention, and data collection methods, but recruited only 19 participants. This illustrates the importance of undertaking feasibility studies prior to fully powered RCTs. From the qualitative interviews we found that potential recruits were not at equipoise for recruitment. An alternative methodology, for example a preference RCT recruiting from multiple locations, needs to be explored in future trials.

**Trial registration:**

International Standard Randomized Controlled Trial Number: ISRCTN31789096

**Electronic supplementary material:**

The online version of this article (10.1186/s40814-018-0303-7) contains supplementary material, which is available to authorized users.

## Background

The World Health Organization estimates that the harmful use of alcohol is a causal factor in more than 200 disease and injury conditions, leading to around 3.3 million deaths every year worldwide, representing 5.9% of all deaths [1].

In England, around 20% of the population drinks heavily: 16.6% at hazardous levels, also known as ‘increasing risk’ drinking; 1.9% at harmful or ‘higher risk’ levels; whereas 1.4% are physically dependent on alcohol [2, 3]. Hazardous alcohol consumption has been defined as a level of consumption or pattern of drinking that is likely to result in harm should present drinking habits persist [4], while harmful drinking is defined as a pattern of drinking that causes damage to health, either physical (such as liver cirrhosis) or mental (such as depression secondary to alcohol consumption) [5]. In the UK, current guidelines issued by the Chief Medical Officer [6] suggest that both men and women should drink no more than 14 units of alcohol per week (1 unit is equivalent to 8 g of alcohol [7]). The harm from problem alcohol use has been estimated to cost England between £21 billion [8] and £47 billion [9] per year. These costs are attributed mainly to the large number of hazardous and harmful drinkers, defined in the “Methods” section below.

Identification and brief advice (IBA) is crucial to helping people avoid alcohol-related harms and is provided in primary care or A&E departments and other front line organisations [10, 11]. If IBA results in lower levels of consumption, this should translate to reduced NHS spending [12, 13] although the effectiveness of this policy is now being questioned [14].

If people continue to drink heavily despite IBA, they may be referred for specialist alcohol treatment. Local authorities are responsible for commissioning alcohol treatment services from NHS and voluntary sector Community Drug and Alcohol Services (CDAS) [15]. There is not only high demand for these services, but also a large unmet need for treatment for people who do not seek help, mainly due to stigma but also due to difficulties in attending appointments due to work or other commitments, a shortage of services, or confusing care pathways [16–19]. There is a need for alternatives to the standard face-to-face treatment model to help overcome these obstacles.

There is potentially a role for alcohol-specific digital health interventions (DHI). DHI provide online access to information and support to help people self-manage a range of physical and mental health problems [20]. They are private, relatively inexpensive to run and convenient to access whenever the user needs help [21, 22]. Around 90% of the population of Great Britain has access to the Internet [23]. DHI may therefore offer an effective and cost-effective way of treating hazardous and harmful alcohol use [24]. However, the trial data comparing DHI with face-to-face treatment in adults are lacking.

Undertaking ‘gold-standard’ RCTs for alcohol research is complex, for a range of reasons. People with alcohol problems are reluctant to participate in trials due to perceived stigma and, if they do take part, may drop out of treatment and the trial itself due to relapse [25–29]. Other well-established reasons for failure to recruit include low numbers of eligible participants, difficulty describing the interventions involved, and complex trial designs or materials [30, 31]. Reasons for participant drop-out also include problems with trial design, particularly the use of long or intrusive questionnaires for outcome measures [32].

It is therefore advisable to carry out feasibility studies for complex interventions and studies before undertaking large-scale effectiveness RCTs and associated studies to understand the challenges of implementation in routine settings. In an earlier mixed methods feasibility RCT, we attempted to recruit hazardous and harmful drinkers attending Community Drug and Alcohol Services (CDAS) for their first appointment, with a view to randomising participants to an alcohol treatment digital health intervention or treatment as usual (TAU). This study was co-designed with alcohol service commissioners who were under the impression that the services saw large numbers of hazardous and harmful drinkers attended these services and so would provide sufficient potential recruits. However, we found that only 5.1% of the CDAS clients were eligible for the trial, as most clients were either dependent on alcohol or had complex co-existing problems such as substance misuse, severe mental health problems, or outstanding criminal justice or child protection issues. Of the minority who were eligible, very few (10.9%) were willing to take part in the study, and retention was poor. We undertook qualitative interviews with alcohol counsellors, to try to explain the quantitative data and understand the barriers and facilitators of taking part [33]. We found that the low recruitment rate was due to both client and counsellor factors. For example, a lack of equipoise may have led to an unwillingness by clients to be randomised. Counsellors were also reluctant to recruit eligible patients to the study, as they were concerned that some clients would not manage an online treatment.

In view of these findings and after further consideration of the policy question faced by commissioners—namely whether scarce resources should be expended on alcohol-related DHI for hazardous and harmful drinkers—we developed the current study. We targeted hazardous and harmful drinkers who had not yet committed to face-to-face treatment, based on our hypothesis that they might be more at equipoise with being randomised to either face-to-face or on-line treatment, having not sought any treatment previously.

We chose to recruit from hospital emergency departments (ED) because large numbers of patients attend with alcohol-related conditions. For example, a 1998 study by Waller et al. found an estimated 35% of ED attendees did so for conditions related to alcohol use, and in 2005, Drummond et al. showed this proportion rose up to 70% at peak times (both studies cited in Drummond et al. [25]). In addition, at the time of the study, EDs were receiving Commissioning for Quality and Innovation (CQUIN) funding [34] to undertake identification and brief advice for problem alcohol use. We aimed to recruit from five north London Hospital EDs. We also decided to advertise the trial online, as previous trials of alcohol DHI have successfully recruited using this strategy [24, 35, 36].

In summary, in the light of our own and other researchers’ well-documented problems with recruitment and retention in alcohol trials, we decided to undertake another feasibility study before attempting a definitive phase 3 RCT to determine the relative effectiveness and cost-effectiveness of treatment delivered face-to-face or through a DHI for hazardous and harmful drinkers.

### Aims and objectives

The overall aim of this study was to determine whether it would be feasible to undertake a definitive trial comparing the effectiveness and cost-effectiveness of a DHI with face to face treatment for alcohol misuse among hazardous and harmful drinkers presenting to hospital EDs or seeking help for alcohol use online.

The specific objectives [37] were:

1. To determine potential recruitment rates to a definitive RCT.
2. To determine rates of retention to the trial.
3. To test online randomisation and data collection instruments.
4. To collect data to inform sample size calculation for the main RCT.
5. To understand the reasons for any problems with recruitment and retention or use of the DHI.

Objectives 1–4 were addressed through the feasibility RCT, and objective 5 was addressed through qualitative interviews.

## Methods

We conducted a feasibility RCT and embedded process evaluation. The trial processes are described below.

### Patient and public involvement (PPI)

Three patient representatives helped design the study, and two sat on the trial management committee. They also took part in think aloud testing of the DHI, describing their immediate reactions while the researcher observed them using it [38]. In order to engage potential recruits to the study, we sought feedback from two other patient representatives on the recruitment materials, the trial portal and the online recruitment, consent and data collection processes. They logged in to the trial portal with a specific code so that their data were not added to participant data. After viewing all the trial materials and the questionnaires, they recommended simplifying the information in the PIL and consent form. In addition, alcohol commissioners and ED staff gave feedback on the proposed design and suggested ways to improve the trial methods.

### Recruitment

Recruitment for the feasibility study ran from October 2015 for 12 months. After the feasibility trial ended, participants were invited to take part in the process evaluation.

### Participants

#### Inclusion criteria

People aged ≥ 18 years drinking at hazardous and harmful levels (AUDIT score > 8 [39, 40]) were eligible to take part in the trial if they were able to use a computer and did not have any of the exclusion criteria listed below.

#### Exclusion criteria

We excluded dependent drinkers (Leeds Dependence Questionnaire [41] score > 20), people who had serious mental illness (SMI) such as schizophrenia or bipolar disorder, those who were at risk of self-harm or suicide or who were currently undergoing treatment for substance use disorder, anyone who had a serious physical health problem (e.g. liver disease, cardiovascular disease, cancer), people with legal issues likely to lead to imprisonment, anyone who was homeless, had child protection issues, was a victim or perpetrator of domestic violence, also anyone who was pregnant, was not able to speak English, or who was not able to use a computer. We also excluded people who did not live in the catchment area for the CDAS units (based on postcode).

### Settings

#### ED departments

We aimed to recruit from five hospital EDs in north London, all teaching hospitals serving the London boroughs of Barnet, Camden, Islington, Enfield, Haringey, and Westminster (covering a combined population of around 1,716,400 people [42]), with a black and minority ethnic population ranging from 32 to 42%, seeing around between 300 and 600 patients a day [43] and screening between 20 and 60% of attendees. In some of the EDs, alcohol liaison nurses and associated administrators were in post and had agreed to help recruit participants. Unfortunately, as the trial started, the Commissioning for Quality and Innovation (CQUIN) funding was withdrawn and identification and brief advice for problem alcohol use became less of a priority for the EDs and funding for these posts was withdrawn.

Recruitment was ‘active’ for 3 days a week (Thursday, Friday, and Saturday) in one ED that was at the time still undertaking alcohol screening of patients using AUDIT-C [44] scratch cards (with the first three AUDIT questions concerning consumption, for which a positive score is greater than 4). The research associates were given honorary contracts at each hospital in order to screen visitors on behalf of the ED. They followed a script for approaching people in the waiting room with the scratch cards (see Additional file 1: Appendix A). For people who were interested, they distributed the cards, collected them, and scored them, and for people scoring positively, or for those who expressed an interest on behalf of family or friends, the RA described the study in more detail and provided the potential recruit with a trial leaflet which explained the purpose of the trial (see the leaflet in Additional file 2: Appendix B for details). The trial leaflet contained instructions on how to self-recruit by visiting the trial website. The research associate also offered to show potential recruits the website and input their details using a tablet device connected to the Internet via the hospital Wi-Fi. The research associate then gave the scratch cards to ED staff, who could then provide tailored advice to patients depending on individual scores. Recruitment was ‘passive’ in the other EDs using posters and leaflets in the waiting rooms and consulting rooms.

#### Online adverts

The trial was also advertised online: on an alcohol treatment website (Down Your Drink (DYD) [45]), an alcohol screening and brief intervention website commissioned by the local authorities of our recruitment area (Don’t Bottle It Up [46]), two alcohol-specific support websites (Soberistas [47] and Club Soda [48]), a community information and local advertising website (Gumtree [49]), and a website providing advice and support to parents (Netmums [50]). People were able to self-recruit directly through the trial website, reached from links in the adverts.

For both recruitment sources, potential participants accessed the trial website by entering their postcodes, as only people who lived in the catchment areas for the participating CDAS units could take part in case they were randomised to this arm. They entered their email address and were sent a validation email with ID and temporary password, which they could change on logging in. They then completed a consent form and eligibility questionnaires. If eligible, they were directed to complete baseline questionnaires and were then automatically randomised by computer and sent an email with instructions for accessing the relevant intervention. Participants were emailed requests to complete follow-up measure online at 1 and 3 months, with an incentive e-voucher of £10 on completion of the outcome measures at the final follow-up. They then had the option to indicate if they would like to take part in the interview study following the feasibility RCT.

##### Recruitment to the process evaluation

Interviewees were participants in the feasibility RCT. They were invited to take part after completing the final follow-up measures online. The website displayed a screen thanking them for taking part in the feasibility study with a request to click a link to indicate they were interested in taking part in further research. Those participants who responded to the invitations were then emailed a PIL and consent form for the qualitative study by the trial manager and were contacted to arrange a convenient time and date for the interview.

### Intervention

This was an online alcohol treatment programme called HeLP-Alcohol [37]. The programme was developed from an online alcohol treatment programme called Down Your Drink (DYD), which mirrored treatments known to be effective face-to-face at community alcohol services [22, 51, 52]. Participants had the option of viewing a film explaining how to use the intervention before progressing. HeLP-Alcohol had three phases: the first phase was based on motivational interviewing, aiming to encourage the user to reach a considered decision about changing drinking behaviours; the second phase used computerised cognitive behavioural therapy (CCBT) and behavioural self-control techniques to help users cut down; the third phase focused on relapse prevention [22]. HeLP-Alcohol also had an online drink diary, and users could set treatment goals and record their thoughts and feelings in response to the various modules. To maximise flexibility, users were given instructions to work through one module per week and had to complete questionnaires before they were able to move on to the next section, but it was not specified when they should access the website or how long to spend on it. They were able to sign up to receive text message or email prompts (depending on their preference) to maximise engagement with the intervention as prompts have been shown to be effective in other studies [53]. There were films of dramatised case studies for each module to maintain interest and engagement. Information about alternative local and national sources of support was also provided. The participants’ use of the website was automatically logged.

### Comparator

The comparator was TAU (face-to-face) in four CDAS in north London. Each CDAS provided treatment tailored to the client: individual face-to-face sessions with a counsellor, and/or group sessions, with the option of attending complimentary therapies at some services, e.g. yoga or gardening. Some CDAS counsellors saw patients at their GP surgery. As this was a feasibility study rather than an efficacy study, we did not standardise TAU across the services.

### Outcome measures

We followed the CONSORT extension to randomised pilot and feasibility trials [54]. The primary outcomes were feasibility outcomes, and secondary outcomes were data collected from patients.

- Primary outcome measures
  Recruitment as a percentage of eligible patients.
  Retention measured by completeness of online data collection for each arm at baseline and at 1 and 3 months as a percentage of patients randomised, which also gives an indication of acceptability of randomisation and each arm.
- Secondary outcome measures

- The self-report measures for each study arm were collected via online questionnaires at baseline and follow-up and are shown in Table 1.

**Table 1:** Secondary outcome measure questionnaires

Item	Description	Collected at baseline	Collected at 1 month	Collected at 3 months
Demographic characteristics	Age, sex, ethnic group, highest educational attainment and area deprivation (measured by Index of Multiple Deprivation [80])	✓	✗	✗
LDQ	Leeds Dependence Questionnaire, a 10-item questionnaire [81]	✓	✗	✗
Unit consumption of alcohol per week	TOT-AL, an online beverage-specific measure [82] which requires \participants to enter the type and quantity of alcohol drinks consumed on each day of the past week	✓	✓	✓
AUDIT	Alcohol Use Disorders Identification Test [39], a 10-item questionnaire developed by the World Health Organization to identify problem drinking	✓	✗	✓
CORE-10	Clinical Outcomes in Routine Evaluation questionnaire [83], a 10-item questionnaire to measure current psychological global distress score developed and validated as a non-proprietary measure of psychological distress	✓	✗	✓
SCQ-8	Situational Confidence Questionnaire [84], an 8-item questionnaire to measure confidence in avoiding alcohol in a range of situations	✓	✗	✓
CSQ-8	Client Satisfaction Questionnaire, an 8-item questionnaire developed to measure satisfaction with care provided by mental health services [85], and also used for assessing satisfaction with alcohol and other substance misuse programmes	✗	✗	✓
Attendance	Whether participant attended CDAS or used HeLP-Alcohol at 1 month	✗	✓	✗
Adherence to the intervention (for those randomised to this arm),	Measured through automated recording of numbers of log-ins and numbers of pages visited at each log-in	✗	✗	✓
Other sources of support accessed during treatment	Using a drop-down menu of options: group therapy, horticulture; acupuncture, art therapy, other therapies (participant to state in free text)	✗	✓	✓

### Data collection for the feasibility study

#### Recruitment

The numbers of participants who logged on, completed baseline questionnaires, and then were randomised were recorded automatically, along with the data on baseline measures.

#### Retention

Participants were emailed requests to complete follow-up measure online at 1 and 3 months, with the offer of £10 shopping e-voucher for completing the outcome measures at 3 months as this level of incentive has strong evidence of increasing completion rates in trials [55–57]. Retention data were collected automatically from those participants who completed online follow-up measures, and usage data for participants randomised to HeLP-Alcohol were automatically captured by the website.

### Data collection for the process evaluation

The topic guide was developed by FH (Co-PI) and JH (trial manager), both of whom have conducted qualitative interviews in previous studies and have undertaken specific training for this. The questions were agreed through discussion with EM (Co-PI) and FS, both of whom have expertise in qualitative research. A pilot interview was conducted by FH and JH in the university department with one of the participants to test the topic guide and finalise the questions. The topic guide is shown in Additional file 3: Appendix C. Subsequent interviews were conducted either by FH or JH, at each participant’s preferred location: at the participant’s place of work, a local café, at the university, and one took place over the phone. Each interview took up to 1 h and was audio-recorded. All identifying details were removed when the interviews were transcribed, and each participant was assigned a unique number. Recordings were stored digitally on university computers until professionally transcribed and were then deleted. Participants took part in the study in their own time and were offered a token of gratitude (a shopping voucher for £10). None of the participants had met with FH or JH previously, but the names of the trial team were on the recruitment literature so participants might have realised the qualitative researchers were also the trial researchers. When meeting the participants and asking them to sign the consent forms, FH and JH discussed the reasons for the interviews to get feedback on the experience of being in a trial and what insights the participant could bring to improving a future trial.

### Sample size

As this was a feasibility study, there was no formal sample size calculation. Feasibility and pilot studies usually aim to recruit around 70 participants in total for estimation of key parameters [58]. We aimed to interview around 20 trial participants for the embedded process evaluation.

### Analysis of quantitative data

As this was a feasibility study, the outcomes for each arm were described but not compared using statistical methods, although confidence intervals were applied to numerical results for the main outcome measure (past week’s alcohol intake in units).

### Analysis of qualitative data

Interview recordings were transcribed verbatim. Transcripts were coded by hand by FH and JH, having read and reread the transcripts so they were very familiar with the data. Emergent themes were developed, discussed by the team, and agreed by thematic analysis [59]. The team also looked for disconfirming data [60]. EM, FS, FH, and JH are all female researchers at UCL, and all have PhDs. EM and FH are general practitioners, FS is a medical sociologist, and JH is a trial manager.

## Results

### Recruitment and retention

**Fig. 1:**
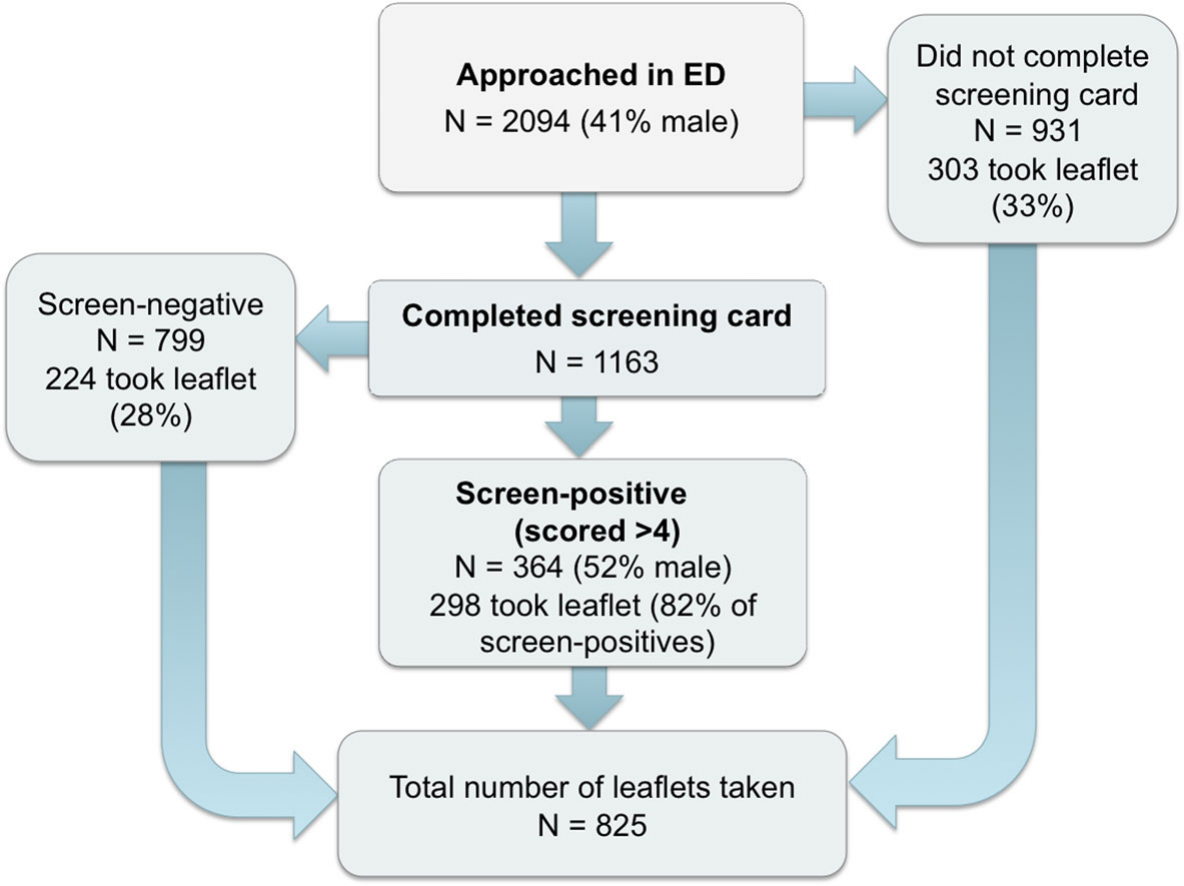
ED recruitment flowchart

**Fig. 2:**
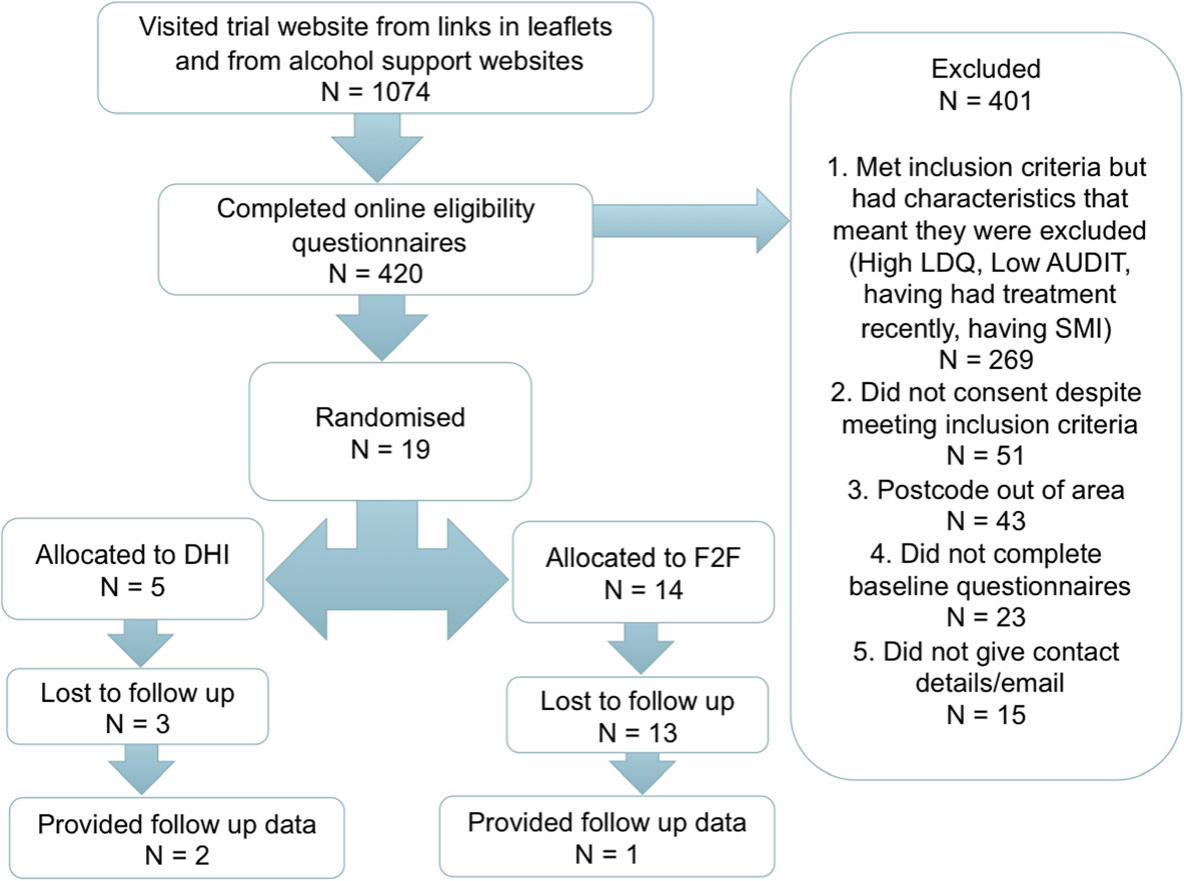
Website recruitment flowchart

Over the course of the trial, 19 participants were recruited, 12 from the websites and 7 from EDs, of whom 2 were from active recruitment. At 3 months, 16 were lost to follow-up, and we collected follow-up data from three trial participants. CONSORT diagrams for recruitment and participant flows are given in Figs. 1 and 2 and a detailed breakdown of the recruitment is described below.

For the 6-months’ active recruitment at ED1, during 34 sessions, each lasting around 6 h in the afternoon and evening on a Thursday, Friday, or Saturday, the RAs approached 2094 people, collected scratch card scores from 1163 patients, of whom 364 were positive (31%), with an average score for positive patients of 7.12 (95% CI 6.83 to 7.41). The RAs handed out 825 trial leaflets, 298 to screen-positive patients (82% of screen-positive patients accepted a leaflet); 224 to screen-negative patients who expressed an interest in finding out about the study; and 303 to people who did not complete the scratch card but said they wanted to pass on the leaflet to a family member or friend who they were concerned was drinking heavily. The main reasons given for screen-positive patients not taking leaflets were that they were either not interested or considered themselves low-level drinkers and did not want help with their drinking.

Overall, there were 1074 visits to the trial website, by people who had linked from the ED trial leaflets or from the online adverts. Four hundred twenty people subsequently logged in to the trial portal using their postcode, of whom 43 people were out of area and so were not eligible to participate, and a further 269 did not fulfil eligibility criteria, e.g. were dependent drinkers or were already in treatment.

In addition to the 19 people randomised, another 51 people completed the eligibility questionnaires and were eligible to take part but either did not consent or did consent but did not complete their baseline measures and so were not randomised. After the end of the recruitment to the feasibility studies, six participants expressed interest in taking part in the qualitative study. All were contacted by email with the PIL and consent form. Of these, four people agreed to take part in the interviews, one from the DHI arm and three from the face-to-face arm, including one participant who had dropped out of the feasibility RCT. We have not given their demographic details in the quotations below to avoid possible identification. We also emailed others who had registered on the trial website but had not completed the baseline measures, as their views would have been helpful, but none responded to the invitation.

### Themes from the process evaluation relating to recruitment and retention

As there were only four interviews, we used a simple descriptive thematic analysis, rather than seeking higher order themes from a realist or critical realist perspective. There were three main themes identified from the four interviews, discussed in more detail below:

1. Reasons for participating in the study;
2. Reasons for not engaging with the intervention to which randomised or for not providing follow-up data; and
3. Changes that could be made to the trial design and intervention.

#### Reasons for participating

The interviewees expressed high levels of motivation to seek help for their alcohol use, and interest in taking part in the trial in order to help other people get evidence-based treatment.

Because I thought my drinking was just normal levels of drinking… it was a bit of an eye-watering thing to know that, like, it was more than what I thought it was. It made me think, got to get my life back on track and cut down on how much I’m drinking. Participant 160802

I think I needed to get it done. I mean, it needed to be done. I, it was getting out of control…and obviously it’s helping people, isn’t it? Participant 160728

#### Reasons for not engaging with the intervention to which randomised or for not providing follow-up data

These included not accepting that they had a drink problem, or not being ready to give up drinking, so this led to disengagement with the trial.

I don’t have a major, major alcohol problem. I just drink a lot of beer, you know... I don’t think I have a problem. Sometimes I think I need to quit or I need to stop drinking, but I don’t know. Participant 160728

Life as a problem drinker interfered with some participants’ ability to take part in the trial or access CDAS, with heavy drinking leading to forgetting appointments. For others, having a busy life and working but being randomised to face-to-face made it difficult to attend CDAS appointments and so led to drop out.

It was, I’ll make an appointment to go down and then... I might have been drinking at the time, so I don’t remember…you know, chunks get lost sometimes. Participant 160801

The participants described not being at equipoise, i.e. they did not feel that both interventions would be equally effective for them [61]. For example if they had experienced face-to-face treatment and wanted to try the website, they were disappointed to be randomised to face-to-face, so did not turn up or only went for one session.

I was quite excited about the idea of being randomised to be online as I’ve had lots of face-to-face therapy over the years and so I was looking forward to a different experience, but I was randomised to face-to-face. Participant 160922

I would have chosen the online based on history of face-to-face therapies, talking therapies if you like, which I haven’t found particularly helpful in the past. Participant 160922

However, this could also be interpreted as being due to a failure of face-to-face treatment, coming from the experience of the interviewee, such that the novelty of the online version appealed to them, without any experience of the this intervention, perhaps more a hope that it would be helpful.

#### Changes that could be made to the trial design and intervention

Participants found data collection repetitive and that the trial processes were sometimes confusing and led to inefficiencies or delays, e.g. the email with the outcome of their randomisation and next steps to take went to their junk folder then they had to contact the research team themselves to find out what was happening. There was a positive response to prompts/reminders by email or text. They also recommended providing an online or app-based drink diary for people in the face-to-face group as well as those in the website group.

If I get people ringing and ringing I get quite anxious… it’s sort of like being stalked. People ignore email and phone calls and voice mail... text would be very good… everyone reads texts. Participant 160801

I think it probably would have been better had I been given more reminders, because it’s, kind of, like, somebody else, kind of, almost subconsciously looking out for me, if that makes sense? Participant 160802

To improve recruitment for a future phase 3 study, participants suggested recruitment from other clinics or GP surgeries with poster or leaflets or invitation from GP, also to recruit from clubs and societies such as the Women’s Institute, political groups, gyms, and online alcohol support groups, particularly for recruiting women.

### Online randomisation and data collection instruments

Online randomisation and data collection were feasible. The HeLP-Alcohol usage data for those randomised to this arm was automatically captured and showed that the median number of pages visited was 23 (range 2–81), and for all participants this was all on the first day of logging on.

### Data to inform sample size calculation for the main RCT

Using last value carried forward for those without follow-up data, the primary outcome (previous week’s alcohol intake in units) reduced as shown in Table 2 below.

**Table 2:** Change in alcohol intake (units/week)

Group	N	Baseline mean (95% CI)	Mean change at 3 months (95% CI)
Face-to-face	14	35.66 (23.91 to 47.41)	− 1.73 (− 4.58 to 1.11)
Help-Alcohol	5	59.23 (3.96 to 114.45)	−8.03 (− 23.58 to 7.51)
Combined	19	41.86 (27.79 to 55.94)	− 3.39 (− 7.14 to 0.36)

For the group randomised to receive F2F treatment, intake reduced by 1.73 units/week (95% CI − 4.58 to 1.11) from a baseline of 35.66 units/week (95% CI 23.91 to 47.41); and for the group randomised to HeLP-Alcohol intake reduced by 8.03 units/week (95% CI −  23.58 to 7.51).

## Discussion

We recruited fewer participants than we hoped to, and fewer than most feasibility trials aim for [58], but this is not unique to our study, as a search of the ORRCA (Online Resource for Recruitment Research in Clinical Trials) [62] database identified other studies aiming to recruit problem drinkers from hospital settings and found low recruitment rates from screen positive patients. For example, a 1999 RCT in which nurses were trained to recruit problem drinkers from the ED had to be abandoned due to low recruitment rates [63]. We also recruited low numbers of participants in a previous feasibility study conducted in community alcohol services [33]. In that setting, the low recruitment was due to the services seeing mainly dependent drinkers, and low numbers of hazardous and harmful drinkers without additional complexities, and lack of equipoise among both potential recruits and alcohol counsellors who were recruiting them. In addition, the exclusion criteria for that study and the current study were rather exacting for safeguarding reasons, as requested by the CDAS units.

As described above, specific CQUIN funding [34] to undertake IBA in EDs was withdrawn just as the trial started, and with it the alcohol liaison nurses who were originally going to recruit participants. This meant that recruitment had to be by passive means (posters and leaflets) until further funding was secured for RAs to recruit participants. However, when active recruitment finally went ahead, it appeared to be less successful than the passive approach. This was despite the RAs identifying a high proportion of screen-positive patients, with 30% of those completing a screening card having a positive screening result (a similar finding to Waller et al. as cited in the paper by Drummond et al. [25]) and 82% of screen-positive patients taking a trial leaflet. People may not have wanted to take part in the trial due to the stigma associated with problem alcohol use, or because when people were screened and found out their alcohol intake put them in hazardous or harmful categories, this came as a surprise and they were not at the appropriate stage of change [64] to consider reducing their drinking, much less take part in a trial. Another possibility is that, due to the availability and marketing of alcohol in society, heavy drinking is normalised [65, 66], so hazardous and harmful drinkers do not believe they need any intervention or think that intervention is more appropriated for dependent drinkers [67].

There are potential ways to address the recruitment difficulties we faced. The RAs could have handed out the trial leaflet only, not the alcohol screening card, and the screening could have taken place if potential participants accessed the website. Then brief advice could be provided online for people who screened positive, along with the invitation to take part in the study. This could have set their drinking level in the context of being ‘at risk’ and that treatment could benefit them at this stage rather than people with more established problem drinking. Alternatively, RAs could have been trained to give brief advice for anyone who screened positive, which might have helped frame the potential benefit to participating in the trial. In addition, other successful strategies for recruiting problem drinkers in the ED to RCTs, identified from a search of the ORRCA database, included a 2006 study by Diguiseppi et al. using telephone follow-up to recruit people who screened positive for hazardous drinking to a lifestyle intervention trial [68], and a 2007 study by Graham et al. which found higher rates of recruitment of injured patients in acute care settings using a laptop for screening with AUDIT-C versus paper-based screening thought to be due to reduced social desirability bias [69].

The majority of participants appeared to have signed up as a result of advertising, either through posters and leaflets in A&E or on the alcohol support and community websites. Several more could have been recruited but they lived outside the trial areas, and, while acknowledging that we should have mentioned the area restrictions in our advertising, the response suggests that a phase 3 trial advertising in multiple locations could recruit sufficient numbers of participants. The use of diverse sources of recruitment was successful in a 2009 study by Morley et al. recruiting to their alcohol RCT, albeit for dependent drinkers [70]. However, for that trial, the researchers did not use online recruitment, and it is possible that in our trial potential recruits may have found it confusing to be recruited face-to-face for a trial with an online component or vice-versa, and this may have affected recruitment rates.

Participants had a strong preference for one or other intervention, and this was sufficient to either cause them to decline to participate in the study, resulting in low recruitment numbers or to drop out of the study if not randomised to their treatment of choice. This may also explain the low usage figures for those randomised to HeLP-Alcohol. People were meant to work through one module per week and complete the tasks before moving on to the next section. Evidently most people were flicking through the website rather than engaging with it, so either the website was not engaging or people were not ready to engage with it. These findings agree with those of our previous study [71] and findings of other research regarding participants’ dissatisfaction with the intervention to which they were randomised, for example in their review, Thomson et al. found ‘the most common barriers to patient participation involve fears of assignment to placebo treatment, insufficient compensation and poor attendance at initial appointments’ [72]. The lack of engagement with either face-to-face treatment of DHI has been described as due to the difficulties associated with problem alcohol use itself [73, 74].

Despite the disappointing recruitment figures seen in this study, it is important to continue to try to undertake trials of digital alcohol interventions for hazardous and harmful drinkers. Our study found that participants have a strong preference for treatment arms, and this may be sufficient to affect recruitment and retention rates. For people who have not yet sought CDAS treatment, a preference for DHI may reflect the need for anonymity that other researchers have identified when recruiting to alcohol studies [75, 76]. A recently published survey study of 438 Soberistas.com members found that only half had ever sought face-to-face help for their drinking and that they value the ‘convenience and anonymity’ of the website as the reason for their continued membership [75]. This suggests that a preference RCT design may overcome the recruitment difficulties of the first study [77, 78]. For such a study, we would also aim to maximise equipoise by providing online feedback messages at randomisation: to explain why the randomisation is important, how participants will benefit from the intervention to which they are randomised, how they will be helped, and offer participants the alternative group at the end of the study.

### Strengths and weaknesses

In this feasibility trial, we were able to trial a number of recruitment approaches and explore facilitators and barriers to recruitment in subsequent qualitative interviews. We struggled to recruit from ED using active methods, although advertising online and via posters and leaflets in ED showed more promise, limited mainly by the catchment areas of the associated CDAS, suggesting that a larger study recruiting from multiple locations may be more successful. The trial retention rate was also low. Although we enlisted patient representatives to trial the website and recruitment materials, and received positive feedback from them, we acknowledge that self-selected patient representatives are often different from the target population [79]. Although we enlisted the help of five patient representatives, they could not possibly represent the diverse range of people who would make up our target users. They may have been better educated or have less chaotic lives, and what might have been acceptable for them might not have been acceptable to other problem drinkers and this may have contributed to the poor recruitment.

The qualitative study provided insights into the difficulties that alcohol trials often encounter with recruitment and retention due to client-, recruiter-, and system-factors. A potential weakness was that the interviewers were also involved in developing and running the feasibility trial and so the interviewees might have been reluctant to criticise the trial. In addition, we acknowledge that the main weakness of the qualitative study was that we were only able to interview four trial participants and we were not able to interview people who initially expressed interest in the trial, but did not complete baseline measures or provide consent, because they did not respond to repeated email invitations. However, the insights provided from the four interviewees are useful for informing future research.

## Conclusion

This feasibility study was not able to recruit sufficient numbers of participants to achieve its objectives of testing recruitment, retention, and data collection methods. We explored the barriers to recruitment through qualitative interviews and found that preference for intervention arm affected both recruitment and retention, particularly the inconvenience of attending face-to-face appointment, participants’ need for privacy, and the nature of problem alcohol use affected participants’ ability to engage with treatment and the trial itself.

Our findings are important in planning further research to answer the following pressing research questions: how can we improve recruitment and retention to trials comparing alcohol DHIs with face-to-face treatment and how can we improve use of alcohol DHIs? Traditional RCTs may not be suitable for this population. Given the strong treatment preferences expressed by participants, the way forward is likely to be a preference RCT.

## Additional files

Additional file 1: Appendix A Script for Recruitment in Emergency Department. (DOCX 629 kb)

Additional file 2: Appendix B DIAMOND recruitment leaflet. (PDF 1405 kb)

Additional file 3: Appendix C Topic guide for participants in the DIAMOND feasibility trial. (DOCX 88 kb)

## Abbreviations

AUDIT: Alcohol Use Disorders Identification Test
AUDIT-C: Alcohol Use Disorders Identification Test (Consumption)
CDAS: Community Drug and Alcohol Services
CONSORT: Consolidated Standards of Reporting Trials
DHI: Digital health intervention
DIAMOND: Digital Alcohol Management ON Demand
ED: Emergency department
HeLP Alcohol: Healthy Living for People who use Alcohol
IBA: Identification and Brief Advice
NHS: National Health Service
NRES: National Research Ethics Committee
PI: Principal investigator
PIC: Participant Identification Centre
PIL: Participant Information Leaflet
PPI: Patient public involvement
RCT: Randomised controlled trial
SBI: Screening and brief intervention
SMI: Serious mental illness
TAU: Treatment as usual
UK: United Kingdom
WHO: World Health Organization
