# Supplementary material for: DIAMOND (DIgital Alcohol Management ON Demand): a feasibility RCT and embedded process evaluation of a digital health intervention to reduce hazardous and harmful alcohol use recruiting in hospital emergency departments and online
Source: Pilot Feasibility Stud. 2018 Jun 15;4:114. doi: 10.1186/s40814-018-0303-7 (PMC6003139; doi:10.1186/s40814-018-0303-7)

**Appendix A: Script for Recruitment in Emergency Department (ED)**

Number the ‘Re-think your drink’ scratch cards (NHS Change4Life ‘Rethink your drink’ score cards, for a copy see over).

1. At start of shift introduce yourself to the matron in ED (navy blue uniform) & wear ID pass
2. Attend 7.45pm nurses handover meeting in seminar room – introduce yourself and brief trial overview
3. Let staff know when you are leaving at end of shift
4. If not busy in ED visit Ambulatory care for potential participants
5. Additional FAST scratch cards in holders around ED or ask reception staff.

Recruitment Process

1. Ask **all** visitors to ED not just patients.
2. Give them a scratch card and leaflet if they accept.
3. Script:

   “ Hello I’m a member of staff and we are asking all of our visitors to complete a scratch card on how much alcohol they drink, or if you drink any” 

   Give them a scratch card.

   “Once you have answered the questions I will collect your score and please give the card to the doctor when you are seen (for non patients you collect card). If you score over 4 we are running a trial of free online programme to try to help people cut back on drinking alcohol to a healthier level. Here is the leaflet. All information to enrol is on the leaflet and you will receive a voucher for participating”
4. Show website if interested [www.help-alcohol.com](http://www.help-alcohol.com/)  .
5. Complete spreadsheet (record date, time, sex, yes or no to leaflet, reason for not taking leaflet if offered/their comments.

**‘Rethink your drink’ scratchcard**

Front**:**


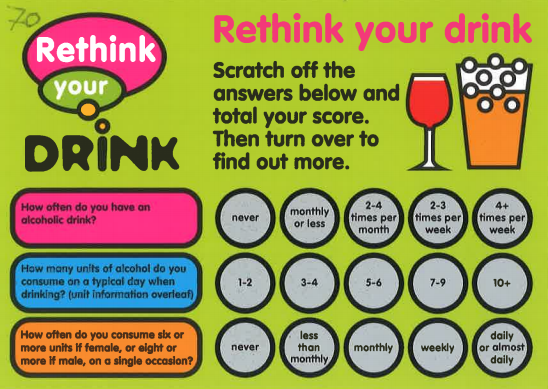


Back


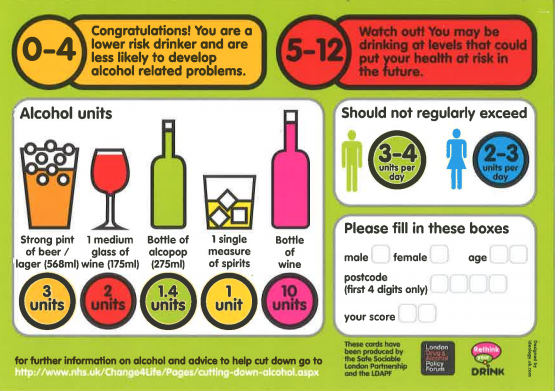

Supplement: Supplementary file 1 — Appendix A Script for Recruitment in Emergency Department. (DOCX 629 kb) [file 40814_2018_303_MOESM1_ESM.docx]
