# Supplementary material for: DIAMOND (DIgital Alcohol Management ON Demand): a feasibility RCT and embedded process evaluation of a digital health intervention to reduce hazardous and harmful alcohol use recruiting in hospital emergency departments and online
Source: Pilot Feasibility Stud. 2018 Jun 15;4:114. doi: 10.1186/s40814-018-0303-7 (PMC6003139; doi:10.1186/s40814-018-0303-7)
Supplement: Supplementary file 2 — Appendix B DIAMOND recruitment leaflet. (PDF 1405 kb) [file 40814_2018_303_MOESM2_ESM.pdf]

DIAMOND Trial

Digital Alcohol Management on Demand

Are you:

- Aged 18 years or over?
- Want help controlling your drinking?
- Able to use a computer?
- Interested in taking part in a trial to test an online service for support, information and tools to control your alcohol intake?

If the answer is **YES** to the above please visit www.help-alcohol.com

NHS
National Institute for Health Research

Version 1.1, 12th June 2015

DIAMOND Trial

Digital Alcohol Management on Demand

- We are inviting you to join a research study, run by UCL and the NHS
- The study aim is to find safe, effective, and acceptable ways of helping people who want help to reduce their drinking, that the NHS can afford
- At the moment there is a shortage of treatment available and we want to find out whether web-based treatment can help people
- If you agree to join the study, you will be allocated by chance (randomised) to either the current standard treatment given in local community alcohol services, or a web-based treatment with additional support
- Whichever treatment you are allocated, it will be up to you to follow up and use the treatment
- We will ask you to complete questions about your experiences and drinking in one month and three months. For more information visit **www.help-alcohol.com**

NHS
National Institute for Health Research

Version 1.1, 12th June 2015
