# Supplementary material for: DIAMOND (DIgital Alcohol Management ON Demand): a feasibility RCT and embedded process evaluation of a digital health intervention to reduce hazardous and harmful alcohol use recruiting in hospital emergency departments and online
Source: Pilot Feasibility Stud. 2018 Jun 15;4:114. doi: 10.1186/s40814-018-0303-7 (PMC6003139; doi:10.1186/s40814-018-0303-7)
Supplement: Supplementary file 3 — Appendix C Topic guide for participants in the DIAMOND feasibility trial. (DOCX 88 kb) [file 40814_2018_303_MOESM3_ESM.docx]

**Appendix C Topic guide for participants in the DIAMOND feasibility trial**

1. **Participant’s attitudes, views and feelings regarding taking part in the DIAMOND feasibility trial**

**Which intervention were you randomised to?**

*How did you feel about being asked to take part in a study where you might be allocated to a website rather than seeing a counsellor for face-to-face therapy?*

*(Prompts if required – some people might feel relieved at the idea of not seeing a counsellor but others might be surprised or offended that they were given the option of a website).*

*Do you think a study like this is useful?*

*(If yes, ask for their experience of being in the trial and how it could be improved; and how we could recruit more people. If no, find out why).*

1. **Data gathering**

*What did you think about completing the questionnaires on line?*

*(Prompts if required: Did you realise the questions were part of the study? Did you understand why you were being asked to complete them? If not, what would have helped with this? Did the systems work? Were the questions easy to understand? Did they seem relevant?).*

*Were there any delays in getting emails or links?*

*How did you feel when you found out which treatment you were randomised to?*

1. **Feelings about using the intervention (website) versus face-to-face treatment**

***For participants randomised to HeLP-Alcohol***

*If you were allocated to the website, how did you feel about this?*

*(What were your initial reactions? Did those change over time? Why?)*

*Did you use the website and why?*

*Did you receive emails, texts or phone calls to support you in using it and if so, were they helpful or not helpful?*

*Did you set up reminder emails or text messages yourself and if so were they helpful or not helpful?*

*Would you have liked to have spoken to a counsellor by phone as well as using the website?*

*What other therapies did you access during the time of your treatment, either through the community alcohol service or things that you found out about yourself?*

***For participants randomised to face-to-face treatment***

*If you were allocated to face-to-face treatment, how did you feel about this?*

*(What were your initial reactions? Did those change over time? Why?)*

*Would you have liked to use the website as well or instead of face-to-face treatment?*

*What other therapies did you access during the time of your treatment, either through the community alcohol service or things that you found out about yourself?*

1. **Did you get the voucher for completing follow up?**

**(before or after completing?)**

1. **Any other thoughts about the trial that we haven’t already covered?**
